# Transsacrococcygeal ganglion impar block

**DOI:** 10.1016/j.inpm.2025.100595

**Published:** 2025-06-09

**Authors:** Kelly Li, David Hao

**Affiliations:** a Harvard Medical School, Boston, MA, USA; b Department of Anesthesia, Critical Care and Pain Medicine, Massachusetts General Hospital, Harvard Medical School, Boston, MA, USA

Abstract

Ganglion impar block is an image-guided procedure used to manage refractory perineal and pelvic pain, often related to malignancy or chronic neuropathic conditions. This educational video demonstrates the transsacrococcygeal approach for ganglion impar block under fluoroscopic guidance. The video reviews indications, relevant anatomy, procedural steps, and potential complications. Key features include identification of the sacrococcygeal junction, needle trajectory planning, confirmation of needle placement with contrast injection, and delivery of local anesthetic or neurolytic agent. This content is intended to supplement formal instruction and enhance understanding of a targeted technique used in the management of complex pelvic and rectal pain.

## Declaration of competing interest

We have no conflicts of interest to report.

## Appendix A Supplementary data

Multimedia component 1 Side-by-side comparison of fluoroscopic image and anatomical illustration showing ganglion impar location and needle positioning

